# Tele-Rehabilitation Systems for Empowering Parents and Their Children with Disabilities in India – A SWOT Analysis of the Context for Implementation

**DOI:** 10.19080/GJIDD.2021.08.555743

**Published:** 2021-07-06

**Authors:** Shobana Devi Moorthy, Manigandan Chockalingam, Sureshkumar Kamalakannan

**Affiliations:** 1 Department for Child Development, Smart Sensory Kids, Chennai, Tamil Nadu, India; 2 School of Health Sciences, National University of Ireland Galway: Galway, Ireland; 3 Research Fellow International Centre for Evidence in Disability, London School of Hygiene and Tropical Medicine; 4 South Asia Center for Disability Inclusive Development and Research, Public Health Foundation of India

**Keywords:** Telerehabilitation, Children with disabilities, India, Disability, Rehabilitation, Health systems

## Abstract

Persons with Disabilities (PWD) experience unmet needs related to health, rehabilitation, education, livelihood, social participation, and empowerment, particularly those living in a resource-poor context such as in the Indian context. The same applies to Children with Disabilities (CWD) as well. Given the pandemic restrictions imposed by the government of India, the provision of therapeutic rehabilitation services for PWDs and CWDs has come to a deadlock. Therefore, the PWDs and the parents of CWDs are substantially impacted by the double contextual burden of demand and access to rehabilitation services in India. However, there has been some light at the end of this dark tunnel provided by the existing telecommunication strategies. Both parents/caregivers and rehabilitation service providers started to find a way out of this situation on their own in India by optimizing their skills and resources for telerehabilitation. However, adopting this strategy requires evidence. Hence a critical Strengths Weaknesses Opportunities and Threats (SWOT) analysis of the telerehabilitation strategy for empowering PWDs and CWDs in an Indian context is warranted and is of immense public health importance. A narrative review was conducted.

Telerehabilitation has several strengths, weaknesses, opportunities, and threats. Telecommunication resources, Access to Rehabilitation services, Parent’s and consumer acceptance, Service efficiency, and data documentation could be considered as strengths; Skills, Competencies, Opportunity cost, Resource intensiveness, Evidence for Effectiveness, Comprehensibility could be considered as weaknesses; Therapy innovations, Evidence generation, System strengthening and Capacity Building could be considered as opportunities; Patient Safety, Ethical Integrity, Data security, and Professional practice insecurity could be considered as potential Threats to Telerehabilitation. Telerehabilitation has considerable scope for providing meaningful therapeutic experience and hastens the process of rehabilitation of CWDs in the current context. The SWOT and its implications must be kept in mind to ensure that CWDs receive the best quality continuum of care in the present context with utmost ethical and evidence-based considerations. This could bridge the gaps in access to rehabilitation services with sustainable solutions than patchy temporary solutions that are not sustainable.

## Abbreviations

PWD: Persons with Disabilities
CWD: Children with Disabilities
GBD: Global Burden of Diseases
WHO: World Health Organisation
COVID-19: Corona Virus Disease 2019
SWOT: Strengths Weaknesses Opportunities and Threats
LMICs: Low and Middle-Income Countries
HICs: High-Income Countries

## Background

Persons with Disabilities (PWD) experience unmet needs related to health, rehabilitation, education, livelihood, social participation, and empowerment, particularly those living in a resource-poor context such as in Indian context [1]. The same applies to Children with Disabilities (CWD) as well1. About 15% of the world population experience some form of disability [2].

However, in India, it is believed to be 2.21% of the population [3]. Although the disability prevalence is relatively lesser, the rehabilitation needs are substantially higher in India [4]. A recent study using the Global Burden of Diseases (GBD) data to assess the rehabilitation needs in BRICS nations revealed an exponential increase in the rehabilitation needs of CWDs in India [5]. Lack of accessibility, availability, and affordability of rehabilitation services as well as the limited resources for rehabilitation are the key reasons for this increase in unmet needs [6]. Even in a scenario that is quite the opposite of the one above, it is understood that the increased demand for rehabilitation services will never cease because of the rapid epidemiologic, demographic, and economic transitions in India [7].

Rehabilitation enables PWDs of all ages to maintain or return to their daily life activities, fulfill meaningful life roles, and maximize their well-being [8]. It is a set of interventions needed when a person is experiencing or is likely to experience limitations in everyday functioning due to aging or a health condition, including chronic diseases or disorders, injuries, and trauma [9]. The role of rehabilitation in ensuring PWDs to be a productive member of their community and in successful integration into their society is well-documented too [10]. Rehabilitation of children with disabilities is even more critical considering the outcomes and its long-term impact [11]. Feasibility for rehabilitation is limited due to various barriers to access rehabilitation services in the country. Nonetheless, 70% of PWDs live in rural areas, thus warranting better rehabilitative solutions and approaches [12].

Given the situation, developing successful rehabilitation models and their appropriate implementation is of paramount importance to meet the current need for rehabilitation [13]. Various successful models for rehabilitation have been proposed and established in developed countries [14]. Adopting such approaches and models in India may not be feasible since the health and social care systems in a developed and developing country like India are very different [15].

One must also not forget that this is a pre-COVID context to empower PWDs and especially CWDs in India. The World Health Organisation (WHO) declared the Corona Virus Disease 2019 (COVID-19) outbreak as a pandemic and advised the call for countries to take immediate actions and response to treat, detect and reduce transmission to save people’s lives [16]. Subsequently, on 22^nd^ March 2020, India announced ‘Janata Curfew’, urging people to stay home except those in essential services extended till 31^st^ May 2020 in four phases [17]. Given the pandemic restrictions imposed by the government of India, the provision of therapeutic rehabilitation services for PWDs and CWDs has come to a deadlock [18]. Rehabilitation service institutes and clinics have been closed since March 20, 2020. Parents could not take their kids to rehabilitation centers or even hospitals because of the pandemic travel ban [18]. Therefore, the PWDs and the parents of CWDs are substantially impacted by the double contextual burden of demand and access to rehabilitation services in India.

Much of the information available to the general public regarding the guidelines to be followed during the pandemic was not available in accessible formats [19]. Even if there is some information provided by civil societies to support PWDs and CWDs, it was not practically possible to be followed by PWDs and CWDs especially if they experience visual, hearing cognitive-perceptual and psychological impairments. The situation is not very different for parents and family caregivers. Taking up a new role of specialized therapy providers at home and abiding by the government imposed pandemic restrictions suddenly is highly challenging and potentially not feasible [20].

However, there has been some light at the end of this dark tunnel provided by the existing telecommunication strategies. Both parents/caregivers and rehabilitation service providers started to find a way out of this situation on their own in India by optimizing their skills and resources for teleconsultations [20]. The consumers and providers of rehabilitation services started to use telecommunication platforms like Mobile Phone Applications, WhatsApp, Skype, Zoom, Google meet to ensure the continuum of care for PWDS and CWDs at their home [21]. This practice has gained momentum since March 2020, and there have been several debates and discussions relating to formalizing this strategy for telerehabilitation in India recently [22]. Hence a critical Strengths Weaknesses Opportunities and Threats (SWOT) analysis of the telerehabilitation strategy for empowering PWDs and CWDs in an Indian context is warranted and is of immense public health importance.

### Tele-rehabilitation for persons with disabilities

Tele-medicine or health is the use of electronic technologies and telecommunications in the field of health [23]. This concept is radically different when it comes to applying telecommunication technologies for the provision of rehabilitation services [24]. Rehabilitation interventions are complex, and it requires different skilled expertise at different points of time and in different intensities for CWDs experiencing disability with varying severity [24].

Tele-rehabilitation is defined as “the clinical application of consultative, preventative, diagnostic, and therapeutic services via two-way interactive telecommunication technology” [25]. Therefore, the primary aim of telerehabilitation could be to minimize the barriers encountered during the provision of rehabilitation services, namely, inaccessibility, unavailability, and non-affordability. Besides it also aims to meet the needs of CWDs through optimizing telecommunication resources [26]. Figure-I depicts the key aspects of telerehabilitation and its inter-connectedness. Rehabilitation professionals like Occupational Therapists (OT), Physiotherapists (PT), Speech and Language Therapist (SLT) who closely work with CWDs can deliver telerehabilitation services to those who could not access these services in-person [26].

## SWOT Analysis of the Context

Given the pre-pandemic and the present pandemic context for rehabilitation service provision, telerehabilitation could be a feasible strategy to meet the needs of CWDs in an Indian context. It could also potentially hold a way for the post-pandemic future for empowering CWDs in India. However, this strategy has several implications for clinical and public health practice which must be carefully analyzed and understood before embarking on accepting it for implementation in a context like India. Figure-II describes the SWOT analysis of the Indian context.

### Strengths

Tele-rehabilitation has several strengths for empowering CWDs in an Indian context. The first and foremost strength is the telecommunication resource itself. India has one of the best telecommunications networks available in the developing world with the promised rollout of 5g connectivity in less than a year [27]. In a context where face to face clinical consultations is restricted, telecommunication technology can connect consumers and providers and create access to rehabilitation services. Given the telecommunication penetration, especially in the country’s rural regions, the distance may no longer be a barrier for CWDs and their caregivers living in rural and remote areas [28]. It would also reduce the substantial cost of travel that the parents of CWDs incur to avail rehabilitation services [28].

Tele-rehabilitation could serve the intended purpose of bridging the gaps in access at a considerably lesser cost and thereby enhancing efficiency in service provision [23]. Telecommunication strategies could also help develop information management systems and make service documentations stored, retrieved, and utilized to impact service efficiency and utilization of the documentation for evidence-based practice and research advancement in rehabilitation sciences [29]. Lastly, the consumers have already accepted this as a better strategy to obtain continued services amidst the pandemic restrictions and pre-existing barriers to accessing rehabilitation services [30]. Parents and caregivers have been using telecommunication technology for personal and professional reasons. They do not find it challenging and unacceptable to use telecommunication to access continued therapy services for their CWDs, especially in this COVID-19 context [30].

### Weaknesses

The weaknesses for implementing telerehabilitation for CWDs in India is just opposite to its strengths. Though telecommunication resources are surplus, India is still a country with a low literacy rate per million population, and this applies to their health, telecommunication, and technology literacy too [31]. The skills and competencies for using telecommunication technologies of the expert rehabilitation service providers are also questionable, mostly because they have not gone through formal training or formally qualified to deliver rehabilitation services through telecommunication technology [32]. The same applies to parents or caregivers of CWDs. They might have the technology but may not comprehend and deliver parent or caregiver-led interventions for CWDs as the rehabilitation experts expect them to.

Most parents may not be prepared for rapid changes in technologies. Furthermore, they find themselves lost and confused about their child’s disabilities [33]. The lockdown situations worsen it further and cause stress within the families. The situation is even more difficult for children as they may not understand the abrupt changes to their routine and this may sometimes be a reason for their disruptive behaviours [34]. Additionally, it may have a tremendous opportunity cost for the parents and caregivers since they might be swopping their scheduled time for employment and other dedicated routines for their time providing telerehabilitation to their children [10].

Remote provision of telerehabilitation services is resource intensive. Resources for telerehabilitation are also prohibitive, which many parents and caregivers of CWDs may not be able to afford [32]. Communication devices such as smartphones, tablets, smart TV, laptops, computers, electronic accessories, adequate internet connectivity, seamless network, and space are required to establish the system both at home and remote rehabilitation center [31–34]. These sources, although available, may not be affordable for many parents and caregivers. Besides the weaknesses, another crucial factor to be considered is the effectiveness of the telerehabilitation interventions. The telerehabilitation effectiveness is neither well established nor researched extensively in the Indian context [35] Although it sounds like a feasible solution, very little research and evidence are available for telerehabilitation in India which could be critical for implementation and scalability [36].

### Opportunities

Reflecting on the weaknesses would portray the potential opportunities that telerehabilitation possesses in an Indian context. Firstly, there is a definitive need to develop innovative interventions that suit the needs of CWDs, the context, the available resources, and the systems [37]. These interventions cannot be prescriptive, and it needs to be systematically developed and tested for its feasibility and acceptability like any other innovations. Once developed, there is a need to establish this innovative telerehabilitation’s effectiveness in an Indian context that could further inform similar contexts, especially in Low and Middle-Income Countries (LMICs). Scientific research providing high-quality evidence on telerehabilitation is the need of the hour for not just India but globally. More research means more evidence to bridge the gaps in rehabilitation service research, which is currently lacking worldwide, especially in terms of relevance more than the scientific rigor.

There are not many systems in place for the organized provision of rehabilitation services both in the government and private sectors. Therefore, the issue of relevance in addition to rigor becomes vital for a context like the Indian context. This relevance is an opportunity for developing appropriate, organized, and inclusive telerehabilitation systems through new systems or appropriate integration in the existing systems [38]. Though lots of work on the clinical aspects for managing disability have been rigorously looked at, it is also critical to strengthen health systems and policy research for rehabilitation within a context like India which is highly relevant to the current demands and needs of CWDs [1].

Lastly, but importantly, to initiate any efforts towards all these opportunities will require capacity. Even to assess the adequacy, acceptance, and feasibility of telerehabilitation innovation, all stakeholders’ capacity building is essential. Thus, capacity building of the rehabilitation service providers, service consumers (parents of CWDs), informal caregivers, telecommunication experts, telerehabilitation administrators, and policymakers on specific aspects and their responsibilities is fundamental. Therefore, it is necessary to have an inbuilt curriculum within the health professional education to build the telerehabilitation capacity for the future [39].

### Threats

Similar to opportunities, there exist few threats too in implementing telerehabilitation in India. A considerable threat that has been spoken about a lot in the recent past has been data theft. Telerehabilitation involves sharing huge volumes of patient information virtually every day. Many clients even make payments online for the services that they may receive. The humongous amount of data generated, utilized, stored, and shared, in day-to-day telerehabilitation practice, may likely be hacked or stolen by hackers and cybercriminals [40]. Therefore, a trusted platform and pathway for information sharing and systems for information management is a must. The second threat comes from the perspectives of providers of rehabilitation services. Existing evidence reports that telerehabilitation can only be an adjunct to in-person rehabilitation [41]. However, rehabilitation service providers feel threatened that telerehabilitation might replace the actual in-person therapy and service provision. This skepticism comes primarily from the choices that parents and caregivers make in deciding to seek therapy services online or in person [32,41]. Given that most of the therapy costs are borne by parents and caregivers of CWDs in an Indian context, they are the actual commissioners for the service providers, and they might decide to go virtual substantially [21].

Ethical integrity could be at threat primarily because of the above two reasons. Lack of trustworthiness in the majority of the existing telerehabilitation platforms, the confidentiality breach, and concern for privacy during the care and rehabilitation of CWDs are a significant threat [42]. Also, there are no pathways for ethical implementation of telerehabilitation, any regulatory frameworks or systems recommended by national or state level authorities, even before the pandemic. Hence no one holds responsibility if/when something goes wrong while providing and seeking therapy services. In the absence of any regulatory framework, it is impossible to take practical actions to streamline practice and against those found guilty of unethical practice. The risks versus benefits also are not made aware to the consumers. This lack of awareness has also created a dilemma among all stakeholders for deciding on telerehabilitation’s implementation strategies. One must ensure telerehabilitation is sought and provided with due ethical considerations [32]. Finally, an important aspect that could be a threat during the implementation of telerehabilitation for CWDs is patient safety [43]. A new technology, new intervention, new experience of therapy service provision and utilization, and a new approach to empowering CWDs can have more risks than benefits. There are several safety implications when it comes to the provision of therapy services using telecommunication technology. Especially the approach to using telecommunication technology to advise therapy procedures. The way the guidelines are interpreted and understood and provided through parent or caregiver-led interventions. There must be an absolute priority that must be provided to patient safety when implementing telerehabilitation for CWDs.

## Implications for implementation of telerehabilitation in India

The “New Normal” way of living in the COVID-19 pandemic has mostly changed people’s lives and livelihood and perspective. However, when it comes to CWDs, even the new normal, is quite different. The “New Normal” has a different “New” and a different “Normal” for CWDs, their parent, and their caregivers particularly in a context like India. The pandemic situation is the only burden for people without disabilities, whereas, for those PWDs and CWDs, it adds to the pre-pandemic burden. The current time may be the ideal time to implement telerehabilitation, considering the “New Normal” way of living.

Telerehabilitation, similar to any other intervention strategies has its strengths, weaknesses, opportunities, and threats. The key consideration in its implementation, however, is reliant on the “context”. The individual, social, cultural, political, and policy situation in a specific country. The understanding of the health professionals and rehabilitation experts about teamwork in the rehabilitation of CWDs, particularly in India is limited since rehabilitation is generally uni-disciplinary, specialist-driven, and available only in urban regions for those who can afford it. The opportunities provided by the “New Normal” paves the way for the much-needed changes in the rehabilitation care delivery both by the rehabilitation providers and the recipients. Currently, the application of telecommunication technology for sustainable development is growing at a significant pace; however, their use in health and rehabilitation is not to that extent. Although very limited, there exist systems in the country funded by the state, private sector, and civil societies to promote the provision of rehabilitation services for CWDs and empower them inclusively. These are considered necessary in High-Income Countries (HICs), and there is a considerable investment in the above aspects in HICs. However, the context overall is very different not just in India but in many LMICs.

Telecommunication technology is compelling in terms of creating an impact if optimized systematically. Experts in the field of rehabilitation must innovate safe, efficient, and effective interventions using telecommunication technology systematically [44]. By being systematic, the authors refer to the appropriate use of MRC guidelines and recommendations for developing complex interventions in health and rehabilitation. Ever since rehabilitation was initiated in India, the experts have only prescribed interventions based on their expertise [6]. In India, the concept of engaging together with interdisciplinary experts to understand the consumers’ needs and systematically develop complex interventions is only developing at best [6]. This practice of limited consultation among experts has several implications in developing relevant telerehabilitation interventions too. For example, technology experts are innovating several technology-driven interventions on the one hand. However, the rehabilitation experts, on the other hand, are not aware of these innovations, and even if they are aware, they do not critically appraise these innovations rigorously for ensuring relevance, thus hampering the use of technology in rehabilitation. Therefore, the telerehabilitation innovations must have inputs from the interdisciplinary team of experts, including telecommunication technological sciences from the early stages of their development, and have suitable protocols to translate technological advancements to rehabilitation providers.

Lastly, CWDs must never be forgotten in technological innovations and the systematic interdisciplinary efforts for telerehabilitation. Two key aspects to keep in mind when innovating and implementing telerehabilitation services are 1. Early and Intensive multi-disciplinary therapeutic simulation could help hasten prognosis and enable them to become independent and empowered. 2. Each child is very different, and therefore the one size fits all kind of intervention does not work when telerehabilitation innovations for children with disabilities are envisaged. This becomes even more challenging for telerehabilitation service providers when impairments and disabilities are severe. A strategic solution to address these key aspects is to firmly adhere to the ethical principles for professional telerehabilitation practice. Telerehabilitation must not harm or outweigh the benefits with its risks and be the source of therapeutic hazards while CWDs receive it. Everyone involved in the provision of telerehabilitation, including parents and caregivers, must hold responsibility for the provision of telerehabilitation services to CWDs.

## Conclusion

Overall, telerehabilitation has considerable scope for providing meaningful therapeutic experience and hasten the process of rehabilitation of CWDs in the current context. The SWOT and its implications must be kept in mind to ensure that CWDs receive the best quality continuum of care in the present context with utmost ethical and evidence-based considerations. This could bridge the gaps in access to rehabilitation services with sustainable solutions than patchy temporary solutions that are not sustainable.

## Figures and Tables

**Figure 1:**
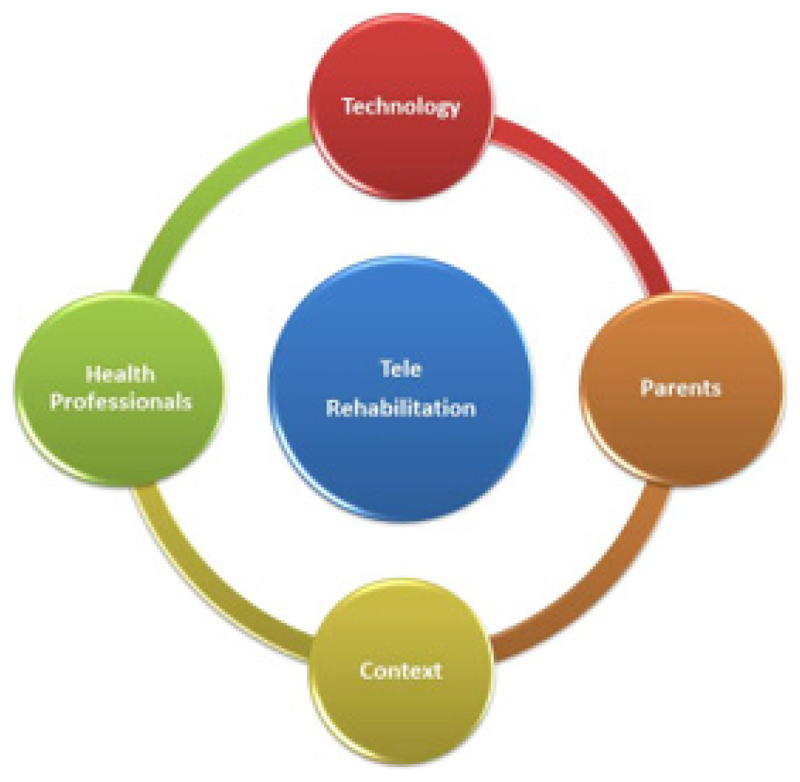
Key aspects for telerehabilitation and its inter-connectedness.

**Figure 2:**
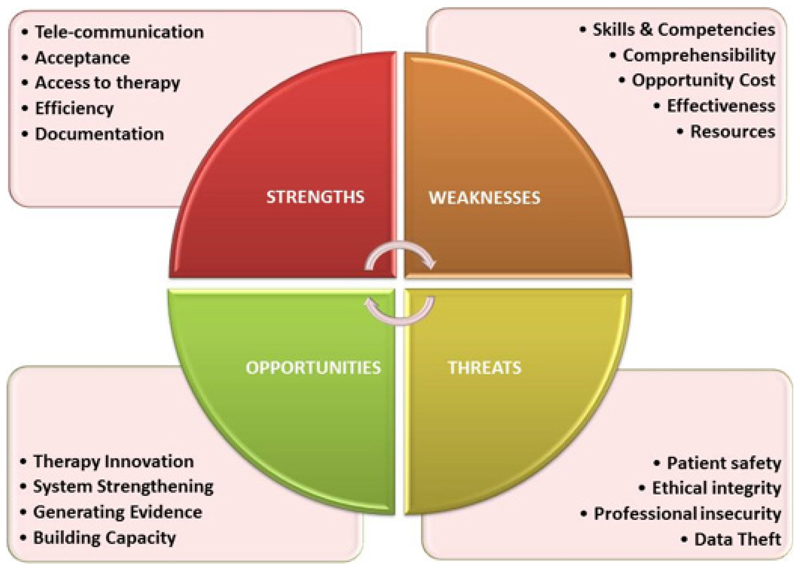
A SWOT analysis of the context for Tele-rehabilitation of CWDs in India.
